# The role of pro-inflammatory S100A9 in Alzheimer’s disease amyloid-neuroinflammatory cascade

**DOI:** 10.1007/s00401-013-1208-4

**Published:** 2013-11-16

**Authors:** Chao Wang, Alexey G. Klechikov, Anna L. Gharibyan, Sebastian K. T. S. Wärmländer, Jüri Jarvet, Lina Zhao, Xueen Jia, S. K. Shankar, Anders Olofsson, Thomas Brännström, Yuguang Mu, Astrid Gräslund, Ludmilla A. Morozova-Roche

**Affiliations:** 1 Department of Medical Biochemistry and Biophysics, Umeå University, 90187 Umeå, Sweden; 2 Department of Biochemistry and Biophysics, Stockholm University, 10691 Stockholm, Sweden; 3 Human Brain Tissue Repository, Department of Neuropathology, National Institute of Mental Health and Neurosciences, Bangalore, 560029 India; 4 Department of Medical Biosciences, Umeå University, 90187 Umeå, Sweden; 5 School of Biological Sciences, Nanyang Technological University, 60 Nanyang Drive, Singapore, Singapore; 6 National Institute of Chemical Physics and Biophysics, Akadeemia tee 23, 12618 Tallinn, Estonia

**Keywords:** Aβ, Alzheimer’s disease, Amyloid, Cytotoxicity, Neuroinflammation, S100A9, Traumatic brain injury

## Abstract

Pro-inflammatory S100A9 protein is increasingly recognized as an important contributor to inflammation-related neurodegeneration. Here, we provide insights into S100A9 specific mechanisms of action in Alzheimer’s disease (AD). Due to its inherent amyloidogenicity S100A9 contributes to amyloid plaque formation together with Aβ. In traumatic brain injury (TBI) S100A9 itself rapidly forms amyloid plaques, which were reactive with oligomer-specific antibodies, but not with Aβ and amyloid fibrillar antibodies. They may serve as precursor-plaques for AD, implicating TBI as an AD risk factor. S100A9 was observed in some hippocampal and cortical neurons in TBI, AD and non-demented aging. In vitro S100A9 forms neurotoxic linear and annular amyloids resembling Aβ protofilaments. S100A9 amyloid cytotoxicity and native S100A9 pro-inflammatory signaling can be mitigated by its co-aggregation with Aβ, which results in a variety of micron-scale amyloid complexes. NMR and molecular docking demonstrated transient interactions between native S100A9 and Aβ. Thus, abundantly present in AD brain pro-inflammatory S100A9, possessing also intrinsic amyloidogenic properties and ability to modulate Aβ aggregation, can serve as a link between the AD amyloid and neuroinflammatory cascades and as a prospective therapeutic target.

**Electronic supplementary material:**

The online version of this article (doi:10.1007/s00401-013-1208-4) contains supplementary material, which is available to authorized users.

## Introduction

The amyloid cascade with the leading role of amyloid-β (Aβ) peptides remains a central concept of Alzheimer’s disease (AD) pathology. AD brains contain soluble (neurotoxic oligomers) and insoluble (plaques) assemblies of Aβ, both of which are the focus of intensive research with the aim of using them as targets for potential therapeutic intervention and raising the possibility that their elimination will lead to an AD cure [36, 44]. Yet the causes triggering Aβ aberrant accumulation in relatively late age still remain elusive and a second causal event may be required to turn on this pathological cascade. Chronic or acute inflammation, associated for example with traumatic brain injury (TBI), may fulfill this function and currently they are the subject of increasing attention [3, 14]. The role of inflammation in AD is supported by a sharp induction of inflammatory mediators in AD-affected brain [25] as well as by epidemiological and experimental studies demonstrating that non-steroidal anti-inflammatory drugs markedly reduce the age-related prevalence of AD [7, 17]. Moreover, these drugs can slow down amyloid deposition by mechanisms that remain still unknown [7]. In this research, we show that the pro-inflammatory protein S100A9 can serve as a critical link between the amyloid cascade and inflammatory events in AD pathology.

S100A9 acts as a pro-inflammatory mediator and its elevated level was found in many inflammatory conditions, including inflammation-associated AD [37]. A significantly increased microglia expression of S100A9 was observed in the temporal cortex of both familial and sporadic AD cases compared to old and young controls [37]. In this study S100A9-positive glia was associated with Aβ diffuse deposits and plaques. The Western blot analysis of the brain extracts also revealed a significantly elevated level of S100A9 monomers and high molecular weight complexes in AD [37], resembling similar dynamics of Aβ expression in AD [36]. Therefore, the authors raised the question as whether S100A9 is involved in plaque pathology [37]. Kummer and coauthors [23] observed a significantly increased level of S100A9 (which is denoted also as myeloid-related protein Mrp14 [40]) in the brain lysates and cerebrospinal fluid of AD patients compared to age-matched controls and detected S100A9-positive microglial cells in the vicinity of amyloid plaques—thus all data implicated S100A9 as a neuroinflammatory marker of AD. A widespread expression of S100A9 was reported in the brain in malaria [34], cerebral ischemia [30] and TBI [10], where it may initiate sustainable inflammatory responses and perform cytokine-like functions controlling inflammatory responses of other cells. In an AD mice model S100A9 production was induced by both Aβ peptide and the C-terminal fragment of the amyloid precursor protein, while S100A9 knockdown attenuated memory impairment and reduced amyloid plaque burden [16]. In AD APP/PS1 mice S100A9 was found to be up-regulated in microglial cells, while the loss of S100A9 by targeted gene disruption led to reduction of the pro-inflammatory cytokine production with dual consequences [23]. Firstly, reduced inflammation decreased the transcription of BACE1 and BACE2, thus limiting the Aβ production and its consequent deposition. Secondly, prevention of excessive microglial activation favored amyloid degradation by microglial phagocytosis, thus acting to protect against AD pathology.

In our previous studies, we have found that the rise of S100A9 level during inflammation may lead to its amyloid formation and deposition in the aging prostate [45] and in the yeast cell model [11]. Both granulocyte extracted and recombinant S100A8/S100A9 complexes formed amyloid oligomers and fibrils also in vitro and the amyloid propensity analysis of S100A8 and S100A9 amino acid sequences revealed that they are intrinsically amyloidogenic [45]. Generally amyloid propensity of S100 protein family was related to their intrinsically disordered sequences, which can be exposed upon loss of structural protection in the native tertiary or quaternary folds and become accessible to amyloid-competent conformations [6]. It has been shown that in vitro the mixture of monomeric and dimeric S100A9 may also induce Aβ fibrillation, suggesting some interactions between S100A9 monomer/dimer and Aβ aggregates, though these interactions remained elusive [47]. Such interactions ultimately reduced the cytotoxicity attributed in this study to non-aggregated S100A9 [47]. The contacts between S100A9 and short Aβ(12–24) peptide, corresponding to the hydrophobic core of full-length Aβ peptides, were analyzed with an atomic resolution by replica exchange molecular dynamics simulation [48]. It was demonstrated that the hydrogen bonding between S100A9 and the ends of this core Aβ peptide induced Aβ(12–24) straightening and consequently promoted its oligomerization. Interestingly, in vitro amyloid fibrillation was found also to be a property of another protein from S100 family—S100A6, as well as its seeding capacity for superoxide dismutase-1 aggregation, which may be related to the amylotropic lateral sclerosis pathology [4].

It is important to note that recently the abundance of S100A9 mRNA was identified as a strong feature of aging in various mammalian tissues, including the central nervous system, and a novel mechanism of age-associated inflammation sustained by S100A9 was suggested [38]. However, the specific role of S100A9 in AD as well as in aging is still far from clear. Here, by using combined analysis of ex vivo AD-affected brain tissues and modeling S100A9 aggregation and co-aggregation with Aβ in vitro, we demonstrate the direct involvement of S100A9 in AD basic mechanisms from the perspective of its intrinsic amyloidogenicity, ability to form plaques and neurotoxicity, thus bridging the AD neuroinflammatory and amyloid cascades.

## Materials and methods

### Materials

Aβ(1–40) and Aβ(1–42) (Alexotech, Sweden) were used in all experiments. Both peptides were dissolved in 10 mM NaOH to avoid aggregation and then diluted into phosphate buffered saline (PBS—140 mM NaCl, 2.7 mM KCl, 10 mM Na_2_HPO_4_ and 2 mM KH_2_PO_4_) 10 mM PBS, pH 7.4 to the required final concentration, determined by Bradford assay and by absorbance at 220 nm [2]. This preparation method, described in more detail in [42], yields highly uniform and reproducible samples well suited for NMR and other spectroscopic and aggregation studies [12]. S100A9 was expressed in *E coli* and purified as described previously [41]. Its concentration was determined by using *ε*_280_ = 0.53 (mg/ml)^−1^cm^−1^.

### Tissue samples

All experimental procedures with tissue samples were approved by the medical ethics committees of the Umeå University Hospital, Sweden, and by the Human Brain Tissue Repository for Neurobiological Studies, National Institute of Mental Health and Neurosciences, Bangalore, India. Brain tissues from seven AD patients with the Braak and Braak [5] stages from III to VI, two severe TBI patients of 35 and 51 years, deceased within 72 h after an accident, and two non-demented control patients—a 75-year-old female, deceased from pulmonary embolism, and a 67-year-old female, deceased from coronary infarction, were examined. All tissues were from the temporal lobe, including the hippocampus, the dentate gyrus, subiculum and also the neocortex. They were paraffin-embedded and microtome-sectioned to 7-μm-thick slices.

### Immunohistochemistry

Single and sequential immunohistochemistry on the same tissue sections was performed as described previously [15] with some modifications [29]. The following antibodies were used: Aβ (rabbit polyclonal, AS08 328, 1 in 200, Agrisea), S100A9 (rabbit polyclonal, sc-20173, 1 in 100, Santa Cruz Biotechnology), S100B (mouse monoclonal, 9A11B9, 1 in 100, Santa Cruz Biotechnology), phosphorylated-tau (mouse monoclonal, AT8, 1 in 25, Thermo Scientific), fibrillar and A11 (rabbit polyclonal, 1 in 200, gift from Kayed [21]), NeuN (mouse monoclonal, MAB377, 1 in 100, Millipore), GFAP (chicken polyclonal, astrocyte marker ab4674, 1 in 500, Abcam), goat anti-chicken IgY (ab97135, 1 in 2000, Abcam), anti-mouse (MP-7402) and anti-rabbit IgG peroxidase reagent kits (MP-7401), Vector Laboratories. The tissues were scanned by a Pannoramic SCAN slide scanner 250 (3D Histech).

### Atomic force microscopy

Atomic force microscopy (AFM) imaging was carried out by a BioScope Catalyst AFM (Bruker) in peak force mode in air at a resonance frequency of ca. 70 kHz and a resolution of 256 × 256 pixels; scan sizes were from 0.5 to 10 μm. Amyloid samples were deposited on the surface of freshly cleaved mica (Ted Pella) for 15 min, washed 3× with 100 μl deionized water and dried at room temperature.

### Fluorescence assays

Thioflavin T (ThT) and 1-anilinonaphthalene-8-sulfonic acid (ANS) assays were carried out as described previously [45] by using a FP 6500 spectrofluorometer (Jasco) with excitation at 440 and 365 nm and detecting emission at 485 and 470 nm, respectively; 5 nm excitation and emission slits were used in both cases.

### Cell culture experiments

SH-SY5Y neuroblastoma cells were cultured with and without amyloids as described previously [20]. Cell viability was measured by WST-1 assay (Roche) after 24 h co-incubation with amyloids. Absorbance at 450 nm was measured by a plate reader (Tecan). Cell viability was expressed as a percentage of the absorbance in wells containing treated cells compared to those of control untreated cells. The amyloid samples prepared for cytotoxicity experiments were lyophilised and reconstituted directly prior to adding to the cells in PBS and added to cell culture media at the required concentration. The morphology of amyloid structures was not affected by lyophilisation as examined by AFM imaging.

### NMR

NMR measurements were performed on a Bruker Avance 500 MHz spectrometer equipped with a triple-resonance Bruker CryoProbe including a *z*-axis gradient coil. Diffusion measurements on a 100 μM S100A9 sample in 10 mM PBS buffer (pH 7.4; 10/90 H_2_O/D_2_O) were carried out at 25 °C using a bipolar gradient routine (Bruker pulse program ledbpgppr2s, using 32 linearly spaced gradient strengths from 5 to 95 % of maximum strength, with 100 ms longitudinal storage time and 2 ms bipolar gradients). The diffusion data for the methyl peak at 0.91 ppm were processed and fitted within the Topspin T1/T2 analysis module. The resulting diffusion coefficient was converted into a corresponding hydrodynamic radius by using the Stokes–Einstein equation. One-dimensional ^1^H and two-dimensional ^1^H-^15^N heteronuclear single quantum coherence (HSQC) spectra of 75 μM ^15^N-labeled Aβ in 10 mM PBS (pH 7.3; 90/10 H_2_O/D_2_O) were recorded at 25 °C both before and after addition of 75 μM S100A9. The assignment of Aβ(1–40) amide peaks was performed previously [9]. The weighted average of the ^1^H and ^15^N chemical shift difference (Δδ = (0.5[Δδ(^1^H)^2^ + (0.2Δδ (^15^N))^2^])^1/2^) and the ratio of amide peak intensities measured before and after addition of S100A9 were calculated.

### Dynamic light scattering

Samples were analyzed on a Zetasizer Nano (Malvem) DLS instrument by scattered light at 630 nm and under 173° angle. 50 μM S100A9 in 400 μl PBS was subjected to five repetitive measurements. Hydrodynamic diameter was evaluated from the autocorrelation curves fitted with the manufacturer’s software and molecular weight was calculated by the Protein Utilities program provided by the manufacturer.

### Molecular docking

The S100A9 dimer [18] and Aβ(1–40) monomer [39] were taken as the receptor and ligand, respectively. The Patchdock [35] and Firedock [1] docking programs were applied to find possible binding modes. 17,316 binary models were generated by Patchdock and further optimized by Firedock. 177 binding modes with a docking score lower than −20 were shown.

## Results

### S100A9 in plaques in the hippocampal and neocortical areas in AD and TBI

**Fig. 1:**
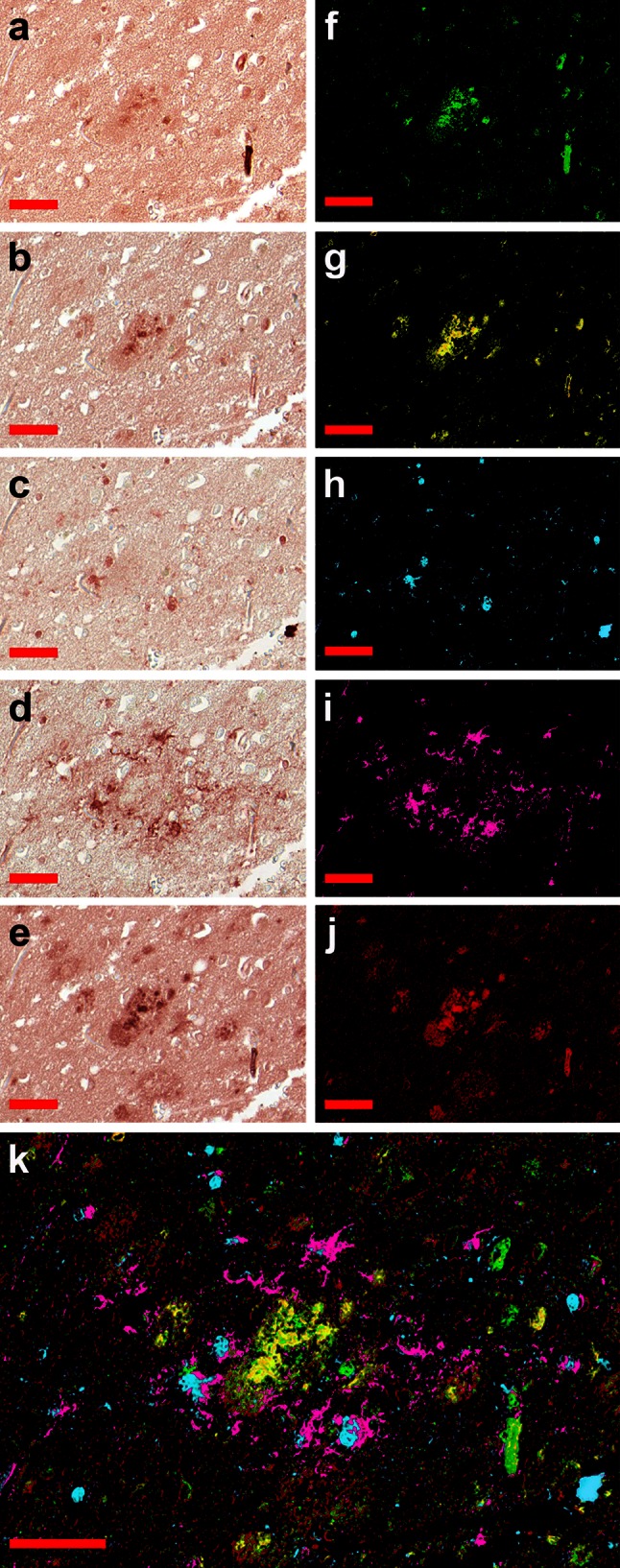
S100A9 and Aβ plaques in the AD hippocampus. Representative sequential immunohistochemistry of the AD hippocampus tissue with **a** S100A9, **b** Aβ, **c** S100B, **d** GFAP and **e** fibril-specific antibodies. The corresponding images are shown in* pseudo-color*: **f** S100A9 staining in *green*; **g** Aβ in *yellow*; **h** S100B in *blue*; **i** GFAP in *magenta* and **j** fibrillar in *red*. **k** Superposition of* pseudo-color* layers. *Scale bars* are 50 μm in all images

**Fig. 2:**
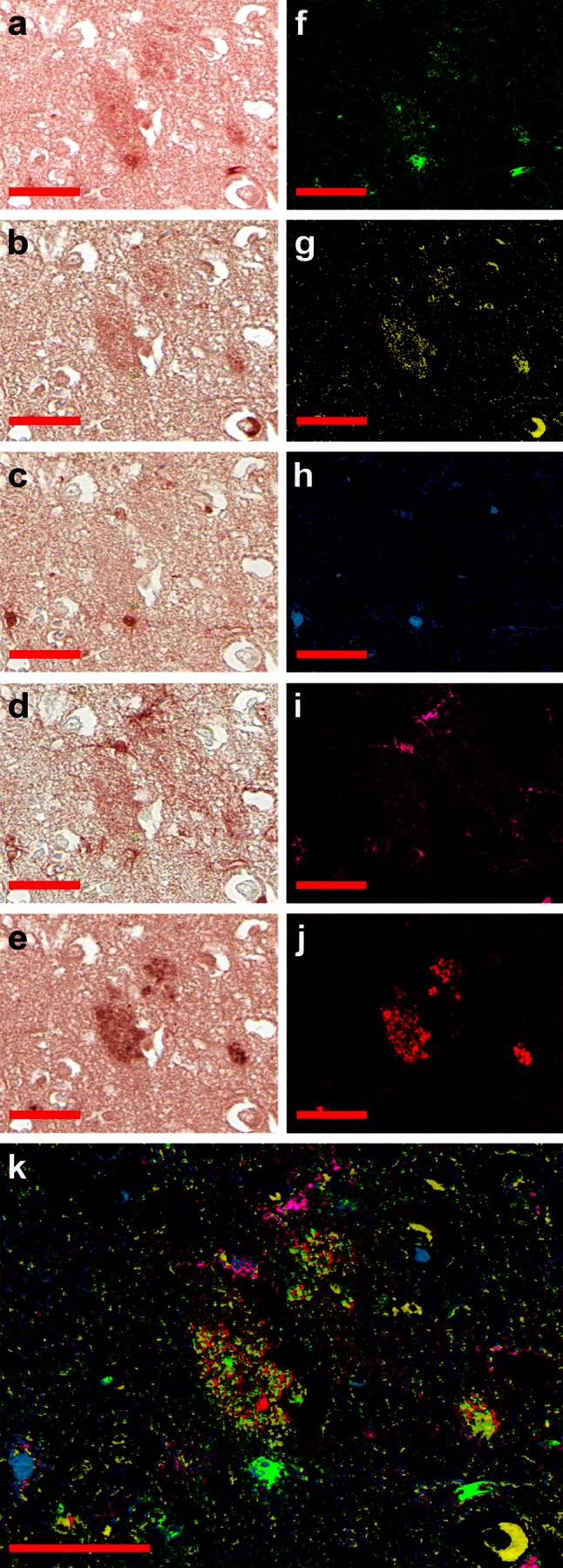
S100A9 and Aβ plaques in the AD neocortex. Representative sequential immunohistochemistry of the AD neocortical tissue with **a** S100A9, **b** Aβ, **c** S100B, **d** GFAP and **e** fibril-specific antibodies. The corresponding images are shown in* pseudo-color*: **f** S100A9 staining in *green*; **g** Aβ in *yellow*; **h** S100B in *blue*; **i** GFAP in *magenta* and **j** fibrillar in *red*. **k** Superposition of* pseudo-color* layers. *Scale bars* are 50 μm in all images

Sections from the AD hippocampi and neocortical areas of seven patients with the Braak and Braak stages from III to VI were analyzed for the distribution and co-localization of pro-inflammatory and amyloid antigens by using sequential immunohistochemistry as shown in the representative images with a higher magnification in Figs. 1, 2 and in the broader low-magnification views in online supplemental files 1 and 2, respectively. We found that S100A9 is abundantly present throughout the whole hippocampi of AD patients, including amyloid plaques, extensive regions around them and blood vessels (Fig. 1a and supplemental file 1a). By contrast, the staining with Aβ antibodies was localized only in the plaques and blood vessels, but not in the surrounding tissues (Fig. 1b and supplemental file 1b). The immunostaining for glial-specific S100B protein was also performed for comparison, as this most studied protein from the S100 family is commonly associated with neurodegenerative disorders and TBI. Interestingly, the staining with S100B antibodies did not overlap with the S100A9 and Aβ patterns: S100B was not detected in plaques and blood vessels, but local staining was observed in tissues surrounding the plaques (Fig. 1c), limited staining was detected around the blood vessel walls and in the white matter of the alveus of hippocampus (supplemental file 1c, low right corner). We have examined the same tissues by immunostaining with GFAP astrocyte-specific antibodies and detected the presence of astrocytes around amyloid plaques. The GFAP-positive pattern showed partial overlap with some local S100B immunostaining (Fig. 1c, d) due to the fact that S100B is produced primarily by mature astrocytes [43]. Staining with anti-amyloid fibrillar antibodies showed a clear correlation with Aβ and S100A9 depositions within the plaques and blood vessels as shown on the individual and overlapping summary images (Fig. 1e, k and supplemental file 1d, i).

Similar to the AD hippocampi, the neocortical areas of AD brains have also shown an abundance of Aβ and S100A9 amyloid plaques, in which the immunostaining pattern for Aβ, S100A9 and fibrillar antibodies were perfectly overlapped (Fig. 2a, b, e and supplemental file 2). Generally, a higher level of S100A9 expression was found also in the surrounding plaque regions, which were not stained, however, with Aβ antibodies. The S100B and GFAP staining was observed in the areas neighboring the plaques, but not in the plaques themselves, with partial overlapping (Fig. 2c, d) as indicated above for the AD hippocampi (Fig. 1c, d). We did not observe any noticeable correlation between the distribution of the Aβ-S100A9 amyloid plaques in the brain tissues and the Braak and Braak stages (III–VI) of AD. The Aβ and/or S100A9 amyloid plaques were not found in the hippocampi and cortical areas of the two non-demented control patients as shown in the representative immunohistochemical staining images for S100A9 in supplemental files 3a, b.

It is important to note that we have also examined the presence of S100A8 plaques in the AD hippocampi and cortical areas, as S100A8 and S100A9 tend to form heterocomplexes in many but not all tissues [13, 40]. We have not found any S100A8-positive plaques in the CA1, CA3, CA4, dental gyrus and neocortical areas (supplemental files 3c–f), while by contrast we have observed the S100A9-positive plaques in all these areas, respectively (supplemental file 3g–j).

**Fig. 3:**
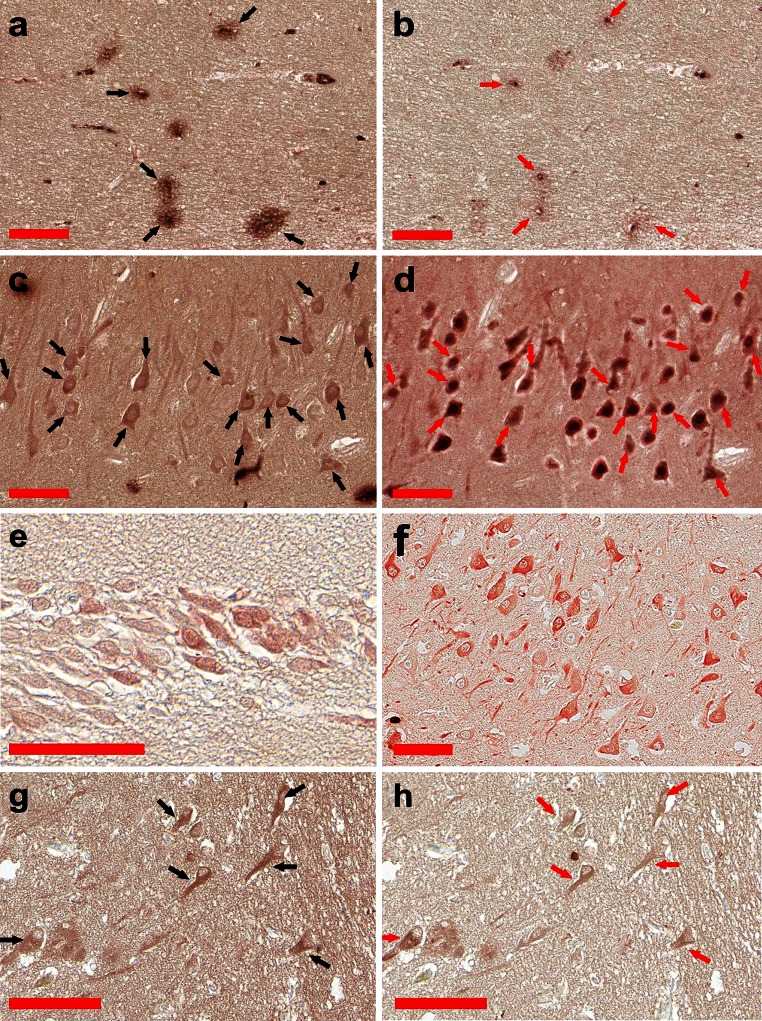
S100A9 in the TBI hippocampal plaques and in TBI, control and AD neurons. **a** Immunohistochemistry of amyloid plaques with S100A9 antibodies and **b** with A11 oligomer-specific antibodies in the TBI hippocampus. **c** Staining of hippocampal neurons with S100A9 and **d** with NeuN neuron-specific antibodies in TBI. **e** Representative staining of neurons in the TBI and **f** the non-demented dentate gyrus with S100A9 antibodies. **g** Representative sequential staining of pyramidal neurons in the CA4 region in AD with S100A9 antibodies and **h** the same neurons were stained with Aβ antibodies. The same plaques and neurons in sequential tissue sections are indicated by *black* (**a**, **c**, **g**) and *red* (**b**, **d**, **h**) *arrows*. *Scale bars* are 100 μm in all images

As TBI is viewed as a risk factor and a potential precursor state for AD, the immunohistochemical analysis for the same antigens were performed on two TBI patients, who deceased within 72 h after the accidents. For both individuals the immunohistochemistry revealed the presence of numerous S100A9 plaques throughout the whole hippocampi (Fig. 3a) as well as their relatively random presence in the neocortical areas close to the hippocampi. By contrast, these plaques were not reactive to antibodies against Aβ, amyloid fibrils and S100B (data not shown), but were stained with A11 anti-amyloid oligomeric antibodies (Fig. 3b). This indicates that S100A9 is not only secreted, but also very rapidly aggregates into amyloid-like plaques in TBI.

### S100A9 in neurons in the hippocampal and neocortical areas in AD and TBI

Apart from the plaques and their surrounding areas, we observed also distinct positive staining with S100A9 antibodies of neuronal cells in the AD, TBI and control aged brain tissues; all S100A9-positive cells displayed specific neuronal morphology. Figure 3c, e demonstrates a representative immunostaining of pyramidal neurons in the CA3 and neurons in the dentate gyrus regions of one TBI patient and similarly the S100A9-positive neurons were observed throughout the whole TBI hippocampus including also the CA1, CA2 and CA4 areas as well as the neocortex. The neurons in the following tissue slice from the same region were also intensively stained with the NeuN neuron-specific antibodies, verifying that these are indeed neuronal cells (Fig. 3d). The S100A9-positive neurons were also observed in the non-demented control patients; the representative staining of granular neurons in the dentate gyrus is shown in Fig. 3f, though the S100A9-positive neurons were observed also in other areas of the hippocampus and neocortex. In the AD patients the S100A9-positive neurons were abundant in all regions of the hippocampus and neocortex as shown in Fig. 3g for the CA4 region and in supplemental file 3g–j for other areas of the hippocampus and neocortex. It is important to note, that some neuronal cells stained with S100A9 antibodies showed also staining with Aβ antibodies, as demonstrated in the representative images in Fig. 3g, h, signifying that S100A9 and Aβ can co-aggregate also within the neuronal cells. By contrast, we have observed an inverse correlation between S100A9 and phosphorylated-tau immunostaining, i.e., neurons intensively stained for S100A9 showed no staining with phosphorylated-tau antibodies (supplemental file 4), indicating that S100A9 is not linked to AD tau-pathology.

### Co-aggregation of S100A9 and Aβ(1–40)

**Fig. 4:**
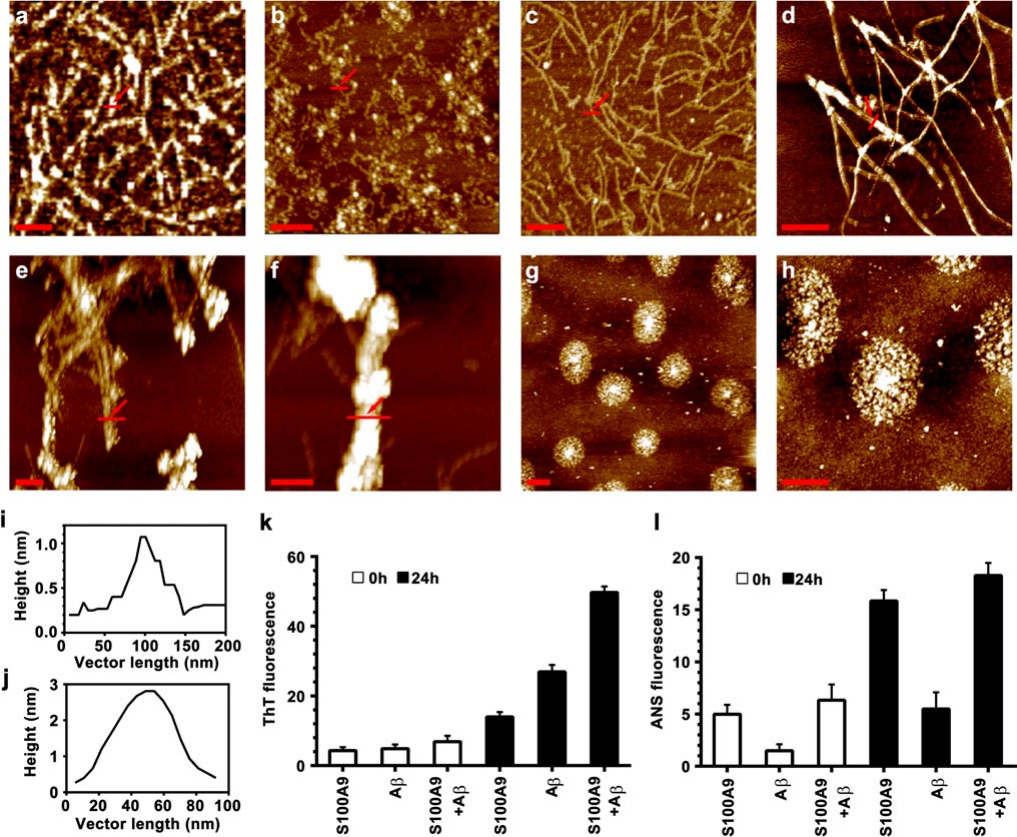
Co-aggregation of S100A9 and Aβ(1–40). **a** AFM height images of S100A9 (50 μM) protofilaments after 24 h incubation in 10 mM PBS, pH 7.4, 37 °C and with 100 rpm shaking; the same conditions were used in all samples, unless specified. **b** AFM image of S100A9 fibrils after 5 days incubation; **c** Aβ(1–40) (20 μM) protofilaments after 24 h; **d** fibrils of co-incubated S100A9-Aβ(1–40) (50 and 20 μM, respectively) after 24 h; **e** coated fibrils of co-incubated S100A9-Aβ(1–40) (both at 20 μM) after 48 h and **f** after 72 h; **g**, **h** the rosettes of co-incubated S100A9-Aβ(1–40) (both at 100 μM) formed after 24 h upon 500 rpm shaking and shown with different magnification; **i** AFM cross-section of representative amyloid filament indicated by *red arrows* in **a**; **f** AFM fibrillar cross-section corresponding to **b**; **k** ThT binding assay; **l** ANS binding assay. In **k**, **l** 50 μM S100A9 and 20 μM Aβ(1–40) were used; *white bars* correspond to freshly dissolved polypeptides and *black bars* to the samples incubated for 24 h. All measurements were referenced to the fluorescence of correspondent free dyes in solution and were taken as an average of five repeats. *x*, *y* *scale bars* are 100 nm (**a**), 500 nm (**b**–**f**) and 1 μm (**g**, **h**)

The amyloid formation of individual Aβ(1–40) and S100A9 as well as their mutual effects on each other were examined in vitro in 10 mM PBS, pH 7.4, 37 °C under 100 rpm shaking (Fig. 4). It has been reported previously that the theoretical amyloid propensity score of S100A9 is close to the propensity of Aβ(1–40) [45]. Indeed, as assessed by AFM imaging, after 24 h incubation S100A9 (50 μM) aggregated into thin protofilaments, which displayed bead-on-string morphology (Fig. 4a); they were less than 1 nm in height as measured by AFM cross-section analysis (Fig. 4i). After 3 days incubation these protofilaments developed into very coily, smooth fibrils (Fig. 4b) of ca. 3 nm height in AFM cross-section and up to a micron length (Fig. 4j). Aβ(1–40) (20 μM) also formed thin protofilaments of ca. 1 nm height with beaded morphology after 24 h incubation (Fig. 4c and supplemental file 5a). By contrast, in the mixture of S100A9 and Aβ(1–40) (50 and 20 μM, respectively), the mature amyloid fibrils were fully developed during the same time period with a thickness of 10 nm and larger and of micron length and longer (Fig. 4d and supplemental file 5b). This clearly demonstrates that S100A9 and Aβ(1–40) drastically enhance each other’s amyloidogenicity.

It is important to note that similar fibrils were developed when Aβ(1–40) (20 μM) was added to pre-incubated S100A9 (50 μM) protofilaments (Fig. 4a) and subjected to further incubation for 24 h (supplemental files 5c, d). This indicates that preformed amyloid aggregates of S100A9 can serve as templating surfaces for Aβ(1–40) amyloid assembly.

When S100A9-Aβ(1–40) co-aggregation (20 μM each) was monitored on a longer time scale, the mature fibrils with ca. 10 nm height were assembled after 24 h (supplemental files 5e, h), then they developed further into massive fibrillar bundles decorated by globular species after 48 h (Fig. 4e and supplemental file 5 g) and subsequently into super-bundles with ca. 80 nm height, as measured by AFM cross-section, after 72 h (Fig. 4f and supplemental file 5 h). With a higher content of S100A9 (100 μM) in the S100A9-Aβ(1–40) solution, even larger aggregated clumps were formed on the amyloid scaffolding (supplemental file 5i). We have reported previously that the S100A8/A9 complexes form super-fibrillar calcified bundles in the aging prostate able to withstand its protease-rich environment [45]. Similarly, S100A9 and Aβ(1–40) can also promote each other’s amyloid assembly into joint large super-complexes. It is important to note, that Aβ(1–40) alone, incubated even at 100 μM concentration for 24 h and up to 7 days, formed mature fibrils but they were still less thick, with ca. 6 nm height in AFM cross-sections (supplemental files 5j, k), and remained smooth compared to the coated fibrillar bundles of S100A9-Aβ(1–40) discussed above.

A drastically different morphology of S100A9-Aβ(1–40) complexes was observed when their concentration was increased to 100 μM each and the shaking rate was raised to 500 rpm. Then S100A9-Aβ(1–40) formed highly populated, very regular, rosette-like structures of up to 2 μm in diameter and with 10 nm height in the center (Fig. 4g, h and supplemental file 5l). This demonstrates that if protein–protein interactions become more favorable due to higher concentration and higher rate of diffusion, the radial nucleation and growth can kinetically out-compete the linear fibrillar assembly.

The ThT fluorescence assay, reflecting specific binding of ThT dye to cross-β-sheet containing amyloids, showed a pronounced increase of ThT fluorescence in all 24-h incubated amyloid samples of S100A9, Aβ(1–40) and S100A9-Aβ(1–40), compared to freshly dissolved polypeptides. The highest ThT fluorescence signal was observed in the S100A9-Aβ(1–40) solution reflecting the development of large quantities of mature fibrils (Fig. 4k).

Protein surface hydrophobicity was examined by ANS binding assay monitoring the increase of dye fluorescence upon binding to hydrophobic surfaces (Fig. 4l). Both freshly dissolved and amyloid species of S100A9 were characterized by significantly larger hydrophobic surfaces than the respective Aβ(1–40) samples, indicating that in the S100A9-Aβ(1–40) mixture S100A9 may be a major hydrophobic contributor. This suggests that S100A9 amyloids may provide very effective hydrophobic interfaces significantly promoting the fibrillation of Aβ(1–40).

### Cytotoxicity of S100A9 and Aβ(1–40) amyloids

**Fig. 5:**
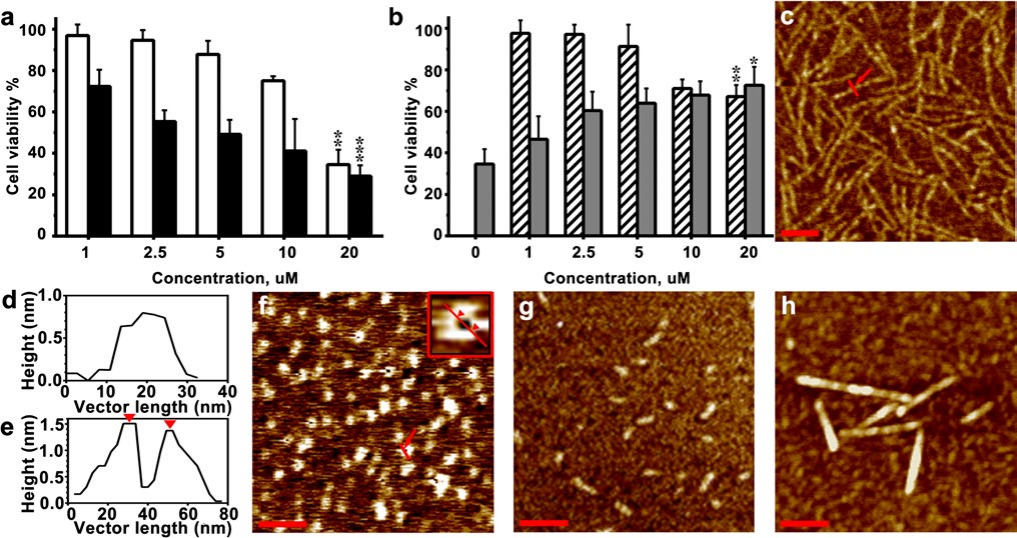
Cytotoxicity of S100A9-Aβ(1–40) amyloids. **a** Viability of SH-SY5Y neuroblastoma cells measured by WST-1 assay after 24 h co-incubation with S100A9 amyloids. The viability of untreated cells is taken as 100 %. *White bars* correspond to cells treated with S100A9 linear protofilaments, *black bars*—with S100A9 annular protofilaments. Bulk S100A9 concentration in μM is shown along *x*-axis. **b** Viability of SH-SY5Y cells measured by WST-1 assay after 24 h co-incubation with Aβ(1–40) and S100A9-Aβ(1–40) amyloids. *Dashed bars* correspond to cells treated with Aβ(1–40) amyloids and *grey bars*—with co-aggregated S100A9-Aβ(1–40). *Grey bar* at zero concentration corresponds to the viability in the presence of 20 μM S100A9 amyloids as in **a**. Bulk Aβ(1–40) concentration in μM is shown along *x*-axis. S100A9 was present at 20 μM in all co-incubated S100A9-Aβ(1–40) samples. *p = 0.04, **p = 0.0016, ***p = 0.0003. **c** AFM height image of S100A9 (20 μM) protofilaments assembled in polypropylene tubes and subjected to cytotoxicity assay after 24 h incubation in 10 mM PBS, pH 7.4, 37 °C without shaking; the same incubation conditions were used in other samples. **d** AFM cross-section of amyloid protofilament indicated by *red arrows* in **c** and **e** AFM cross-section corresponding to **f** image. **f** AFM height image of S100A9 (20 μM) rings formed in more hydrophilic polyacrylic tubes; insertion of 80 × 80 nm shows an individual S100A9 ring. **g** AFM height image of Aβ(1–40) (20 μM) protofilaments and **h** S100A9-Aβ(1–40) (both at 20 μM) amyloid aggregates. *x*, *y* *scale bars* are 100 nm in all images

Since S100A9 is abundant within plaques and hippocampal tissues, we have assessed the cytotoxic damage which S100A9 amyloids can inflict on SH-SY5Y neuroblastoma cells (Fig. 5). The incubation of SH-SY5Y cells with preformed S100A9 linear and annular protofilaments led to a concentration-dependant decrease in cell viability to the level of ca. 35 and 29 %, respectively, at final added polypeptide concentration (Fig. 5a). By contrast, pre-aggregated Aβ(1–40) caused a less pronounced cell viability decline, i.e., to ca. 67 % level at the highest added concentration (Fig. 5b). Moreover, the addition of S100A9 (20 μM) co-incubated with increasing concentration of Aβ(1–40) (from 1 to 20 μM), led to an Aβ(1–40) concentration-dependent increase of cell viability from the level of 35 % (S100A9 alone) to ca. 72 % [S100A9 co-incubated with 20 μM Aβ(1–40)]. It is important to note, that freshly dissolved S100A9 was not toxic at 1–2.5 μM, but displayed ca. 18 % toxicity after 24 h co-incubation with SH-SY5Y cells at 5–20 μM concentrations, possibly due to the on-going protein aggregation under the conditions of the experiments. Specifically, examined here linear (ca. 1 nm and less in height) and annular protofilaments of S100A9 were produced upon 24 h incubation in 10 mM PBS, pH 7.4, 37 °C without shaking (Fig. 5c, d, f). The rings were highly populated upon incubation in less hydrophobic polyacrylic tubes, although they were also present in smaller quantities together with linear protofilaments in four repetitive preparations. They were characterized by ca. 1–1.2 nm height and ca. 20 nm diameter measured between highest points on their circumferences in AFM cross-sections (Fig. 5e) and closely resembled amyloid pores of Aβ and α-synuclein reported previously [24]. The Aβ(1–40) amyloid protofilaments (ca. 2 nm height) and thicker S100A9-Aβ(1–40) fibrils (ca. 4–5 nm height) were produced under the same conditions and are presented in Fig. 5g, h, respectively. As the co-incubation of S100A9 and Aβ(1–40) leads to formation of thicker mature fibrils and potentially even larger aggregates, as we discussed above (Fig. 4), these effectively mitigate the higher cytotoxicity of S100A9 protofilaments (Fig. 5b).

### Co-aggregation and cytotoxicity of S100A9 and Aβ(1–42) amyloids

**Fig. 6:**
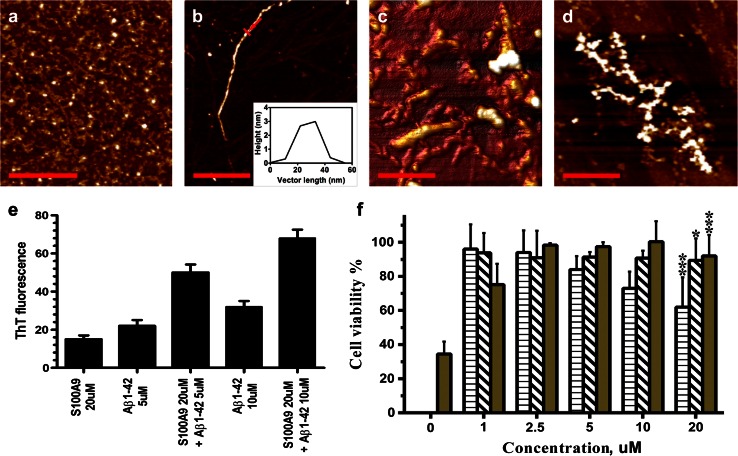
Co-aggregation and cytotoxicity of S100A9 and Aβ(1–42) amyloids. **a** AFM height images of Aβ(1–42) (10 μM) oligomers and protofilaments formed after 7 h incubation in 10 mM PBS, pH 7.4, 37 °C without shaking. The same conditions were used in all samples, unless specified. **b** AFM height image of Aβ(1–42) (20 μM) fibril after 24 h incubation; insertion shows the fibrillar height in the AFM cross-section indicated by *red arrow*. **c** AFM 3D image of co-aggregated “coated” fibrils of Aβ(1–42)-S100A9 (2.5 and 20 μM, respectively) produced after 24 h. **d** AFM height images of co-aggregates of Aβ(1–42)-S100A9 (10 and 20 μM, respectively) persisted after 48 h co-incubation. **e** ThT binding assay for 5 and 10 μM Aβ(1–42) and 20 μM S100A9, respectively, incubated for 24 h. All measurements were referenced to the fluorescence of correspondent free dyes in solution and were taken as an average of three repeats. Specific samples are indicated along *x*-axis. *x*, *y* *scale bars* are 1 μm (**a**), 500 nm (**b**, **d**) and 200 nm (**c**). **f** Viability of SH-SY5Y neuroblastoma cells measured by WST-1 assay after 24 h co-incubation with Aβ(1–42) and S100A9 amyloids. The viability of untreated cells is taken as 100 %. Bulk Aβ(1–42) concentration in μM is shown along *x*-axis. S100A9 was at 20 μM in all experiments. *Olive bars* correspond to cells treated with co-aggregated Aβ(1–42)-S100A9; *bars with horizontal stripe pattern*—to cells treated with Aβ(1–42) oligomers and protofilaments formed after 7 h (Fig. 6a); *bars with diagonal stripe pattern*—to cells treated with Aβ(1–42) mature fibrils formed after 24 h (**b**). *p = 0.026, ***p = 0.0003

We have examined also the co-aggregation and gained cytotoxicity of S100A9 and Aβ(1–42) amyloids, since this peptide together with Aβ(1–40) plays a central role in AD pathogenesis [19]. S100A9 was co-incubated with a range of concentrations of Aβ(1–42) from 1 to 20 μM prior subjecting these species to the cytotoxicity experiments. The cytotoxic samples of Aβ(1–42) developed upon 7 h incubation in 10 mM PBS, pH 7.4 and 37 °C were characterized by the presence of oligomers and thin protofilaments with <1 nm height as shown in Fig. 6a. Upon longer incubation Aβ(1–42) has developed mature fibrils; a representative image is shown in Fig. 6b with a height in the AFM cross-section of ca. 3 nm. By contrast, co-incubation of even 2.5 μM Aβ(1–42) with 20 μM S100A9 led to the formation of much thicker coated fibrils with the variable height in the AFM cross-section from 4–5 to 10–13 nm (Fig. 6c). The same type of coated fibrillar aggregates was observed upon co-incubation of higher concentrations of Aβ(1–42) with S100A9 and persisted on a longer time scale as shown in the representative image of co-aggregation of 10 μM Aβ(1–42) with 20 μM S100A9 after 48 h (Fig. 6d). Similar to the co-aggregation of S100A9 with Aβ(1–40), the complexes of Aβ(1–42) and S100A9 produced at 1:4 and 1:2 molar ratios showed significantly higher ThT signal compared to the individual components incubated during the same period of 24 h (Fig. 6e).

The measurements of the SH-SY5Y cell line viability in the presence of oligomers and protofilaments of Aβ(1–42) showed decrease by up to ca. 40 % upon increasing amyloid concentrations (Fig. 6a, f), while the fibrils of Aβ(1–42) were not toxic (Fig. 6b, f). This is consistent with the previously published results [19]. It is interesting to note, that co-aggregation of S100A9 with Aβ(1–42) leading to the formation of thick coated aggregates resulted in the complete elimination of the Aβ(1–42) and S100A9 amyloid cytotoxicity already at 1:8 molar ratio of Aβ(1–42) and S100A9 (Fig. 6f). This is a significantly more pronounced mitigating effect on S100A9 amyloid cytotoxicity than in the case of co-aggregation of Aβ(1–40) and S100A9, as then even at 1:1 molar ratio the cell viability remained 30 % lower compared to controls (Fig. 5b).

### Interactions of native S100A9 and Aβ

**Fig. 7:**
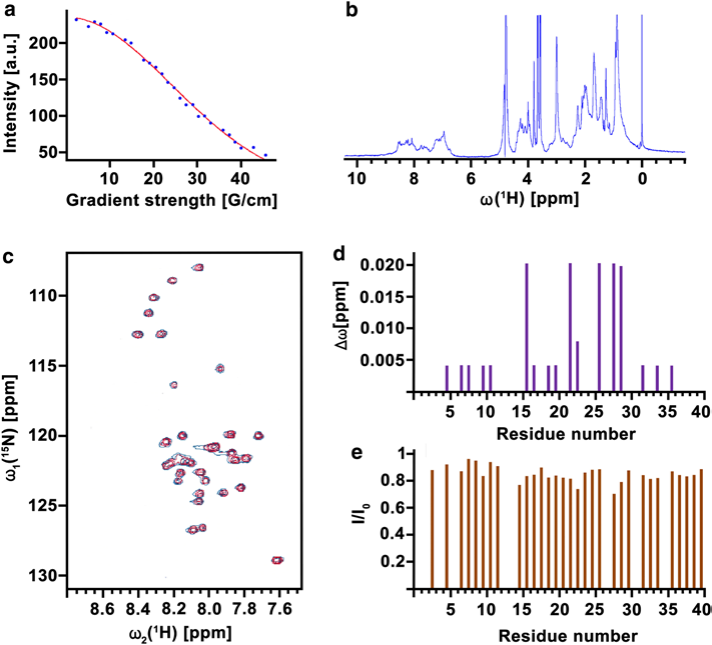
Interaction of native S100A9 and Aβ(1–40) examined by NMR. **a** NMR diffusion of S100A9 measured at 25 °C. Methyl peak intensities at 0.91 ppm in a.u. are shown as a function of gradient field strength *G*, cm^−1^. **b** 1D ^1^H NMR spectrum of S100A9. **c** 2D ^1^H-^15^N HSQC spectra of Aβ(1–40) prior (*blue*) and after (*red*) addition of S100A9. **d** Differences in Aβ(1–40) chemical shifts induced by S100A9 binding versus Aβ(1–40) amino acid residue numbers. **e** Ratios of Aβ(1–40) amide peak intensities measured in the presence (*I*) and in the absence (*I*_o_) of S100A9 versus Aβ(1–40) amino acid residue numbers

The interactions of native S100A9 and Aβ(1–40) (selected as a representative Aβ peptide) were analyzed by using solution NMR and molecular docking. The initial state of S100A9 was evaluated by using NMR diffusion (Fig. 7a) and dynamic light scattering (not shown), which both consistently demonstrated that this is a dimer with a molecular weight of ca. 27 kDa and hydrodynamic radius of ca. 3 nm, respectively. The latter indicated also that ca. 1 % of protein is present in aggregated state. The broad NMR signals in 1D ^1^H NMR spectrum of native S100A9 suggest that there is a chemical exchange and dynamic equilibrium between the monomeric and dimeric forms (Fig. 7b).

2D ^1^H-^15^N HSQC spectra of 75 μM Aβ(1–40) were recorded before and after addition of S100A9. The spectra reflect an essentially monomeric and unstructured form of Aβ(1–40) [42]. Small chemical shifts (ca. 0.015 ppm) for residues K16, E22, S26, K28, G29, together with a 20–30 % decrease in peak intensity for some central Aβ(1–40) residues (e.g., K28), indicate a possible transient binding site in this central region (Fig. 7c–e).

**Fig. 8:**
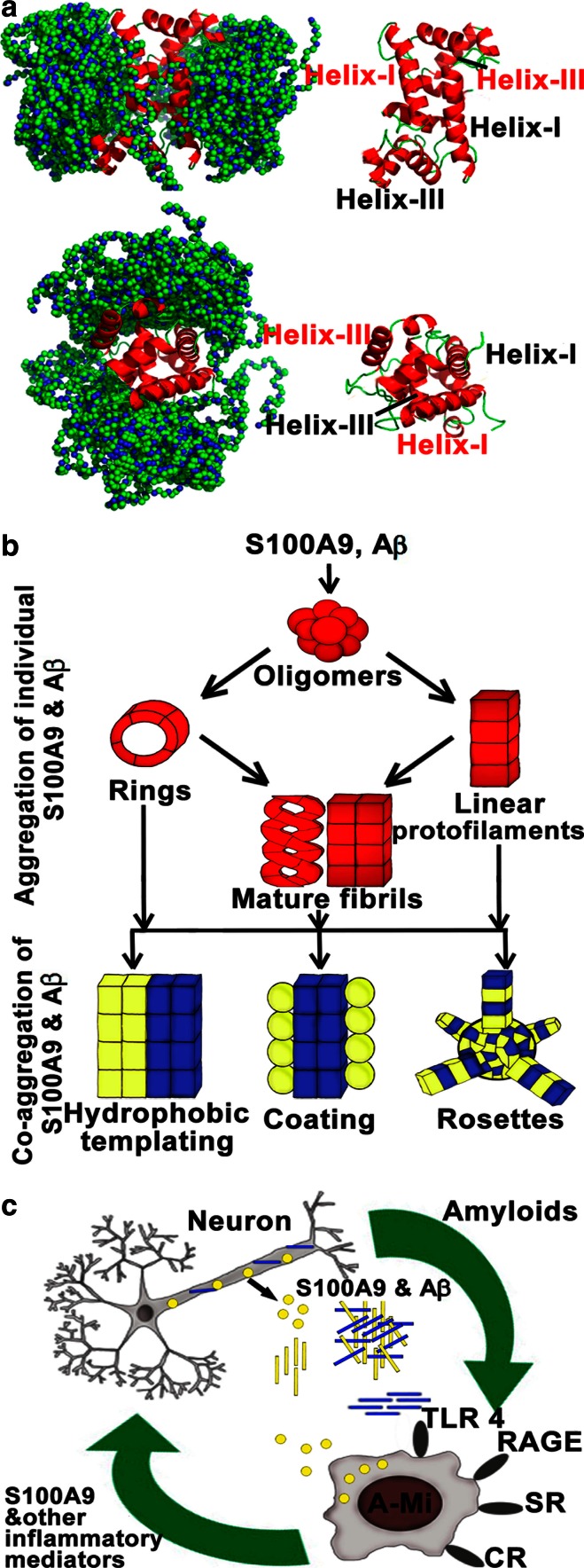
Molecular interactions of S100A9 and Aβ peptides and their role in AD. **a** Molecular docking of S100A9 dimer and Aβ(1–40) monomer. Backbones of S100A9 dimer and Aβ(1–40) are shown in *red ribbons* and *green beads*, respectively. 177 best ranked binding modes of Aβ(1–40) are superimposed. *Upper* and *lower panels* show the side and top views, respectively. Helices I and III as binding candidates are indicated. **b** Schematic summary of multiple amyloid pathways of S100A9, Aβ peptides and co-aggregated S100A9-Aβ. The aggregated structures of individual polypeptides are outlined in *red*; in co-aggregated complexes S100A9 is schematically denoted in *yellow* and Aβ—in *blue*. **c** Schematic outline of the role of S100A9 in AD amyloid-neuroinflammatory cascade. Soluble Aβ peptides (shown in *blue*) and S100A9 (in *yellow*) secreted by neurons and activated microglia (A-Mi) are toxic to neurites and synapses. They accumulate into senile amyloid plaques, which recruit microglial cells. Microglia is further activated by S100A9 via the TLR 4 and RAGE signaling pathways. Level of S100A9 rises, leading to its further amyloid deposition. The cycle is completed as shown by *green arrows*

The analysis of interactions between S100A9 dimer and Aβ(1–40) monomer by molecular docking is shown in Fig. 8a. A total of 177 binding modes of Aβ(1–40) were superimposed on the S100A9 dimer, which were selected based on their docking scores lower than −20. The corresponding binding interfaces involve the two faces of S100A9 dimer, each composed of helix I and helix III′ (helix III′ from the other protomer). Structural clustering analysis for these 177 binding modes with a cutoff of 0.2 nm showed that there are 118 clusters with the largest cluster consisting of 12 members. The multiple clustering without dominant clusters indicates that the binding between S100A9 dimer and Aβ(1–40) has a diffusive character as opposed to specific docking. The S100A9 residues, such as H_2_O from helix I, D30 from the linker between helices I and II and R85 and L86 from helix III, were found to make particularly close contacts with Aβ(1–40). On the Aβ(1–40), residues H14, F20 and K28 from the middle and V39 and V40 from the C-terminus are primary candidates for the interaction with S100A9. This is consistent with NMR results, where 2D ^1^H-^15^N-HSQC measurements showed that S100A9 interactions induce chemical shift changes and reduced signal intensities in central Aβ(1–40) residues, particularly K28 (Fig. 7c–e). Both S100A9 and Aβ(1–40) interfaces and in particular S100A9 helices I and III include significant clustering of positive and negative charges as well as aromatic amino acid residues. Therefore, transient interactions between S100A9 and Aβ(1–40) may include electrostatic component, guiding them towards each other, and hydrophobic binding, providing the interface for Aβ(1–40) molecules diffusion and self-assembly into amyloid structures.

## Discussion

Pro-inflammatory mediator S100A9 was identified as potential contributor to AD development in patients [23, 37] and mice models [16] and to inflammation-dependent aging in the human body [38]. The understanding of S100A9 mechanisms of action is still at a very early stage and this knowledge is critical as S100A9 can serve both as a risk factor and therapeutic target in AD diagnostics and treatment. Indeed, regularly administered general anti-inflammatory drugs, used to treat concomitant diseases, produce also a positive effect on AD, slowing down its progression [7, 26]. Here, we have shown that S100A9 is highly abundant in AD brain (Figs. 1, 2 and supplemental files 1–3), and due to its inherent amyloidogenicity it can play a key role in AD development. Specifically, S100A9 can be a trigger and an important contributor to plaque formation and neurotoxicity. Immunohistochemical analysis demonstrated that together with Aβ peptide S100A9, but not another well-known brain inflammatory protein S100B, is consistently present in the AD plaques and amyloid deposits in the blood vessel walls, forming co-aggregates reactive with anti-fibrillar antibodies. S100A9 is also abundant in tissues surrounding amyloid deposits (Figs. 1, 2 and supplemental files 1–2), indicating that an elevated level of S100A9 can favor its aggregation and deposition.

Remarkably, in TBI hippocampi the numerous plaques that rapidly developed within less than 72 h post-accident were composed of S100A9 on its own, but not with Aβ. These plaques were reactive with A11 amyloid oligomer antibodies, indicating that already within this short time period S100A9 has undergone amyloid-type conformational changes (Fig. 3a, b). It is important to note that apart from catastrophic brain injuries, head traumas are common in various sports and have received increasing attention due to their neuropathological consequences and similarities with AD [3]. TBI is considered also as a potential risk condition for AD development. In AD pathology the amyloid cascade is a central hypothesis and amyloid plaques are a major pathological hallmark of disease, respectively, with the chief role in these phenomena designated to Aβ. We have demonstrated here that highly amyloidogenic S100A9 is able to form amyloid structures in vitro just as rapidly as Aβ (Figs. 4, 5). S100A9 and its amyloids are also more hydrophobic than Aβ (Fig. 4l) and if the plaques fulfill the function of a sink for various toxic species and cell debris not cleared by cellular machineries, S100A9 is a perfect candidate for the role of plaque-forming protein, able to sequestrate all these materials on its sticky hydrophobic surfaces. S100A9 also readily co-aggregates with both Aβ(1–40) and Aβ(1–42) and promotes their amyloid deposition (Figs. 4, 5, 6). Therefore, the amyloid plaques of S100A9 rapidly developed in TBI brain, in fact, can serve as the precursors of AD amyloid plaques, linking TBI and AD via the amyloid cascade mechanism.

S100A9 is secreted by macrophages and the activated microglia in inflammation-associated AD can produce a significantly elevated level of S100A9, which can locally exceed the Aβ level. The initial form of S100A9 is primarily homodimers as we have shown by NMR diffusion experiments and dynamic light scattering, though the broad lines in 1D NMR spectrum indicate that there is an equilibrium exchange between monomers and dimers. However, S100A9 does not stay in its native state for long and within hours or days under close to physiological conditions in vitro (pH 7.4 and 37 °C) it forms a plethora of amyloid complexes, including linear and annular amyloid protofilaments and flexible fibrils (Figs. 4, 5). Remarkably, S100A9 protofilaments and especially its annular species closely resemble similar amyloid structures of leading amyloid polypeptides such as Aβ and α-synuclein in AD and Parkinson’s disease, respectively (Fig. 5, [24]) and they are also cytotoxic towards SH-SY5Y neuroblastoma cells (Fig. 5). However, the toxicity of S100A9 amyloids subsides if S100A9 is pre-incubated with an increasing concentration of Aβ(1–40) and is completely eliminated during co-incubation with Aβ(1–42), starting from their molar ration of 1:8 for Aβ(1–42) and S100A9, respectively. This occurs due to their co-aggregation into larger aggregated species and Aβ(1–42) apparently is more potent in this regard than Aβ(1–40) (Figs. 5, 6). Native freshly dissolved S100A9 by itself also showed some cellular toxicity (ca. 18 %), but due to its progressing aggregation under the conditions of experiment, this toxicity may be related to its aggregates.

Even larger plethora of amyloid species were formed when S100A9 and Aβ(1–40) or Aβ(1–42) were co-aggregated together as shown in Figs. 4, 5, 6 and schematically summarized in Fig. 8b. Since amyloid formation is a concentration-dependent as well as a thermodynamically and kinetically controlled process [28], depending on the concentration ratio of S100A9 and Aβ, one of them can be prompted to aggregate into thermodynamically stable aggregates faster than the other. Here, we consider a few possible scenarios depending on the concentrations of both counterparts and conditions of amyloid formation, such as shaking, which may facilitate protein–protein interactions. As we have shown above, the amyloid fibrils of S100A9-Aβ(1–40) (taken at 50 and 20 μM, respectively) displayed smooth surfaces and were significantly thicker and longer than the fibrils of individual counterparts formed even on a longer time scale and also at higher respective polypeptide concentration (Fig. 4d and supplemental file 5b). Importantly, similar smooth and thick fibrils were produced when preformed S100A9 amyloid protofilaments (Fig. 4a) were added in solution as templates for the Aβ fibrillation (supplemental files 5c, d). Thus, if one of the counterparts is prompted to aggregate faster, for example the more hydrophobic S100A9, it can provide a hydrophobic templating surface for Aβ assembly.

When we mixed S100A9 and Aβ(1–40) at 1:1 ratio and 20 μM each, instead of smooth fibrils, we have observed polymers coated with round-shaped aggregates and the thickness of the coat drastically increased upon 72 h incubation, reaching 80 nm (Fig. 4f and supplemental file 5 h). It is most likely that an Aβ(1–40) fibrillar surface may attract S100A9 coatings, if the former aggregates faster at these concentrations of both counterparts. Similar coating interactions were observed also when S100A9 was co-incubated with Aβ(1–42) (Fig. 6c, d).

Remarkably, when we increased the concentration of both S100A9 and Aβ(1–40) to 100 μM and also introduced vigorous shaking, new rosette-type regular co-aggregates were produced (Fig. 5g). The rosettes were highly populated and constituted the dominant amyloid species in the sample; they reached up to 2 μm in diameter and displayed a regular radial pattern of co-aggregation. This may reflect the fact that the protein binding process is too rapid to be kinetically controlled at more aggregation-prone conditions, thus the radial growth will out-compete the more slow linear assembly. Undoubtedly, all these new types of co-aggregated structures require further structural investigation. Here, our task was to demonstrate their heterogeneity and the possibility of refining their properties by varying incubation conditions. It should be also considered that in the affected tissues the massive and rapid co-aggregation can be a lesser evil as this can effectively remove from circulations more toxic and damaging species. We have shown this above by reducing and eliminating S100A9 amyloid toxicity during its co-aggregation with increasing concentrations of Aβ(1–40) and Aβ(1–42), respectively (Figs. 5b, 6f).

Interestingly, by NMR and molecular docking we observed transient or diffusive interactions between S100A9 dimer and Aβ monomer, but not their specific docking into each other’s surface (Fig. 6a), which would also cause a significant perturbation in the ^1^H-^15^N HSQC spectrum of Aβ (Fig. 5). S100A9 surface hydrophobicity and clustering of charged residues, particularly in helices I and III, provide, most likely, the interface for Aβ own self-assembly, rather than promoting strong binding between Aβ and S100A9. The opposite, e.g., S100A9 self-assembly on Aβ interface, may also take place as we discussed above, depending on the initial conditions and concentrations of both counterparts. Diffusive motility of microtubule and DNA binding proteins may provide an insightful analogue for the nature of the diffusive interactions [8]. The strong specific binding may ultimately lead to inhibition of amyloid formation rather than its promotion as these polypeptides have very different non-complementary amino acid sequences to be integrated within the same cross-β-sheet [22].

Importantly, we have observed also consistent immunostaining of hippocampal neurons with S100A9 antibodies in AD, TBI and non-demented aging, indicating that S100A9 can be also produced by neuronal cells. In some neuronal cells S100A9 was co-localized with Aβ, indicating that their co-aggregation can occur intracellularly (Fig. 3g, h). By contrast, intracellular S100A9 is not co-localized with hypo-phosphorylated tau as it was examined by sequential immunostaining of the same tissues (supplemental file 4), indicating that S100A9 may not be associated with tau-pathology, which is another key feature of AD. It is interesting to note, that overexpression of S100A8 and S100A9 was observed in neurons of Kras mutant mice model acting as an early trigger of gliosis. In the mutant mice cortex the neuronal S100A8/A9 expression pattern was clearly associated with increased infiltration by microglia [32]. It is not excluded that similar events can be also implicated in the inflammatory cascades in AD and TBI. Inflammation clearly occurs in pathologically vulnerable regions of AD brain, but the mediators and mechanisms playing a key role in this process remained as yet obscure. Here, we have demonstrated that S100A9 can act as a link between the amyloid and inflammatory cascades (Fig. 8c).

Indeed, during AD plaque formation microglia become activated and recruited to the plaque deposition sites [31], causing microgliosis. The activated microglia secretes S100A9 as well as an array of other pro- and anti-inflammatory mediators. This can contribute to changes in neuronal calcium homeostasis [27] and accelerate neuritic and synaptic dysfunction [46]. As a result, S100A9 expression in neuronal cells can be also turned on, which further activates microglia via the toll-like receptor 4 (TLR 4) and receptor for advanced glycation end products (RAGE) signaling pathways [32, 33]. A significantly raised level of extracellular S100A9 promotes its amyloid aggregations and also co-aggregation with Aβ. Protofilaments of S100A9 itself are highly neurotoxic (Fig. 5a) and are able to inflict rapid damage and death to neurons. As the co-aggregation of S100A9 with Aβ(1–40) or Aβ(1–42) proceeds more efficiently than for individual counterparts, the co-aggregation process may act as an emergency sink, rapidly removing from circulation the neurotoxic amyloid species of both polypeptides as well as native S100A9 able to act as TLR 4 and RAGE activator. The ultimate price for this “rescue” clearance process, however, can be the exacerbated growth of the amyloid plaques in AD brain. It is known from our previous studies that the amyloid complexes of S100A9 are much more stable and protease resistant compared to amyloids of other proteins [45] and their clearance and removal may be therapeutically unachievable. Furthermore, the plaques themselves may exacerbate microglia activation, thus completing the vicious circle of amyloid-neuroinflammatory cascade (Fig. 8c).

Due to its amyloidogenicity, neurotoxicity and signaling functions, S100A9 may be a promising therapeutic target. Bearing in mind its multifaceted nature, the most effective way would be to target in the first instance its overexpression and secretion rather than deal with subsequent clearance of its multiple native and amyloid species, especially as the latter can be extremely stable and resistant to any interventions. Detailed studies of the effect of non-steroidal anti-inflammatory drugs on S100A9 system, which have already proved to be useful in slowing AD, may hold the key to effective AD treatment.

## Electronic supplementary material

Below is the link to the electronic supplementary material.

**Supplemental file 1** S100A9 and Aβ peptide plaques in the AD hippocampus (broader view). Representative sequential immunohistochemistry of the AD hippocampus tissue with (**a**) S100A9, (**b**) Aβ, (**c**) S100B and (**d**) fibril specific antibodies. The corresponding images are shown in pseudo-color: (**e**) S100A9 staining − in green; (**f**) Aβ − in yellow; (**g**) S100B − in purple and (**h**) fibrillar − in red. (**i**) Superposition of pseudo-color layers. Scale bars are 500 μm in all images.

**Supplemental file 2** S100A9 and Aβ plaques in the AD neocortex (broader view). Representative sequential immunohistochemistry of the AD neocortex tissue with (**a**) S100A9, (**b**) Aβ, (**c**) S100B and (**d**) fibril specific antibodies. The corresponding images are shown in pseudo-color: (**e**) S100A9 staining − in green; (**f**) Aβ − in yellow; (**g**) S100B − in purple and (**h**) fibril specific − in red. (**i**) Superposition of pseudo-color layers. Scale bars are 1 mm in all images.

**Supplemental file 3** S100A8 and S100A9 immunohistochemistry in the AD and control brain tissues. (**a**) Representative images showing the absence of S100A9 positive plaques in the control hippocampus and (**b**) neocortex. Representative images showing the absence of S100A8 positive staining in the AD (**c**) CA1, (**d**) CA3, (**e**) CA4/dental gyrus and (**f**) neocortical regions. S100A9 staining of the plaques and neuronal cells in the AD (**g**) CA3, (**h**) CA4, (**i**) dental gyrus and (**j**) neocortex. Red arrows indicate the S100A9 positive plaques and black arrows – the S100A9 positive neuronal cells. Scales bars are 200 μm in all images.

**Supplemental file 4** Immunohistochemistry of the AD hippocampal neurons with S100A9 and phosphorylated tau antibodies. (**a**) Sequential immunohistochemistry of individual neurons in the AD hippocampus tissue section with S100A9 antibodies; (**b**) with hyper-phosphorylated-tau antibodies; (**c**) with S100A9 antibodies shown in green pseudo-color; (**d**) with hyper-phosphorylated-tau antibodies shown in blue pseudo-color. (**e**) Superposition of pseudo-color layers. Scales bars are 100 μm in all images.

**Supplemental file 5** Aggregation and co-aggregation of S100A9 and Aβ(1-40). (**a**) AFM cross-section analysis of the Aβ(1-40) protofilaments, place of the cross-section is indicated by a red arrow in Fig. 4c. (**b**) AFM cross-section of S100A9-Aβ(1-40) fibrils in Fig. 4d. (**c**) S100A9 seeding of Aβ(1-40) amyloids. AFM height image of S100A9-Aβ(1-40) fibrils formed after 24 h incubation, when 20 μM Aβ(1-40) was added to preformed amyloids of 50 μM S100A9 shown in Fig. 4a. Here and below the incubation conditions were 10 mM PBS, pH 7.4, 37 °C and 100 rpm shaking, unless specified additionally. (**d**) AFM cross-section of amyloid fibril in S100A9-Aβ(1-40) sample, place of the cross-section is indicated by a red arrow in (**c**). (**e**) AFM height image of S100A9-Aβ(1-40) (20 μM each) incubated for 24 h. (**f**) AFM cross-section analysis of representative amyloid fibril in (**e**), place of the cross-section is indicated by a red arrow. (**g**) AFM cross-section of a representative fibrillar bundle of S100A9-Aβ(1-40) in Fig. 4e. (**h**) AFM cross-section of a S100A9-Aβ(1-40) fibrillar bundle in Fig. 4f. (**i**) AFM height image of amyloid clumps of S100A9-Aβ(1-40) (100 μM and 20 μM, respectively) after 24 h incubation. (**j**) AFM height image of amyloid fibrils of 100 μM Aβ(1-40) after 24 h incubation; the same morphology of fibrils remained upon incubation up to 7 days. (**k**) AFM cross-section of a representative Aβ(1-40) fibril indicated by a red arrow in (**j**). (**l**) AFM cross-section of a central S100A9-Aβ(1-40) *rosette* in Fig. 4h. Scale bars are 500 nm in (**c**,**e**,**j**) and 1,000 nm in (**i**).

Supplementary material 1 (TIFF 2503 kb)

Supplementary material 2 (TIFF 1930 kb)

Supplementary material 3 (TIFF 2658 kb)

Supplementary material 4 (TIFF 1840 kb)

Supplementary material 5 (TIFF 1641 kb)
